# Supplementary material for: Microbiota stability in healthy individuals after single-dose lactulose challenge—A randomized controlled study
Source: PLoS One. 2018 Oct 25;13(10):e0206214. doi: 10.1371/journal.pone.0206214 (PMC6201941; doi:10.1371/journal.pone.0206214)
Supplement: S1 File — Detailed protocol of all study procedures including lactulose and sucrose treatment. (DOCX) [file pone.0206214.s001.docx]

| UniversitätsSpital Zürich | u | **Klinik für Gastroenterologie und Hepatologie**  **Funktionsdiagnostik** |
| --- | --- | --- |

Clinical Study Protocol

# Elucidating the influence of lactulose intake on the gut microbiota composition

Short title: Influence of lactulose on gut microbiota

Protocol ID: KEK-ZH-NR. 2014-0358

Version 2.0

17.11.2014

**Sponsor:**

Prof. Dr. med. Dr. phil. Gerhard Rogler

Klinik für Gastroenterologie und Hepatologie

UniversitätsSpital Zürich

Rämistrasse 100

8091 Zürich

E-mail: Gerhard.Rogler@usz.ch

**TABLE OF CONTENT**

1. GENERAL INFORMATION 4

2. STUDY SYNOPSIS 8

2.1. Study Flow chart 9

2.2. List of abbreviations 10

3. INTRODUCTION 10

3.1. Background 10

3.2. Rationale for current study 11

3.3. Aim and significance of the project 11

3.4. Categorization of the study 11

4. STUDY DESIGN 12

4.1. Primary and Secondary objectives 12

4.2. Study procedures, methods outcomes 12

4.3. Method of Randomization and Blinding 13

4.4. Method of encryption 13

4.5. Unblinding Procedures (Code break) 13

5. Recruitment / Participant Entry / Discontinuation 14

5.1. Pre Registration Evaluations 14

5.2. Inclusion criteria 14

5.3. Exclusion criteria 14

5.4. Criteria for discontinuation 15

5.4.1. Voluntary discontinuation by a subject 15

6. Efficacy variables 15

6.1. Demographic measurements 15

6.2. Parameters for assessing protocol compliance 15

7. SAFETY of conducted procedures 17

7.1. Fecal and urine sample collection 17

7.2. Lactulose breath test 17

7.2. Procedures for discontinuation 17

8. STATISTICS AND DATA ANALYSIS 18

8.1. Analysis of fecal and urine samples 18

8.2. Lactulose breath test 18

8.3. Significance level 18

8.4. Dropouts 18

9. Study related regulatory issues 19

9.1. Recruitment and Informed Consent 19

9.2. Confidentiality 19

9.3. Funding 19

9.4. Participant compensation 19

9.5. Preventive measures 19

10. Duties of the principle investigator 20

10.1. GCP 20

10.1.1. Criteria for premature termination of the trial 20

10.1.2. Publication policy 20

10.2. Reporting Procedures 20

10.2.1. Definitions 20

10.2.2. (S)AE recording 21

10.2.3. SAE reporting by the investigator 21

10.2.4. SAE review and reporting by the Sponsor 21

10.2.5. Follow-up of (S)AEs 21

10.3. Insurance 21

11. Ethical considerations 22

11.1. Ethics committee 22

11.2. Confidentiality of study data 22

11.3. Benefits and risks assessment 22

11.3.1. Safety of sample collection and storage 22

11.3.2. Safety of lactulose breath test 23

11.3.3. For Women 23

11.4. Benefits of the current study 23

11.5. Compensation for subjects 23

12. Quality control 23

12.1. Audits 23

12.2. Source data and data encryption 23

12.3. Data handling 24

12.3.1. Documentation and storage 24

12.4. Sample storage and further analysis 24

13. Suitability of the place of inspection 24

13.1. Team 24

13.2. Infrastructure 24

14. References 25

1. GENERAL INFORMATION

**Study title**: Elucidating the influence of lactulose intake on the gut microbiota composition.

**Sponsor:**

Prof. Dr. med. Dr. phil. Gerhard Rogler

Klinik für Gastroenterologie und Hepatologie

UniversitätsSpital Zürich

Rämistrasse 100

8091 Zürich

E-mail: Gerhard.Rogler@usz.ch

**Clinical Investigators**:

**Principal Investigator**:

PD Dr. med. Benjamin Misselwitz

Klinik für Gastroenterologie und Hepatologie

UniversitätsSpital Zürich

Rämistrasse 100

8091 Zürich

E-mail: benjamin.misselwitz@usz.ch

**Additional investigators:**

Prof. Dr. med. Mark Fox, Leiter Funktionslabor

Klinik für Gastroenterologie und Hepatologie

UniversitätsSpital Zürich

Rämistrasse 100

8091 Zürich

E-mail: mark.fox@usz.ch

Dr. med. J. Jonas Zeitz, Assistenzarzt

Klinik für Gastroenterologie und Hepatologie

UniversitätsSpital Zürich

Rämistrasse 100, 8091 Zürich

E-mail: jonas.zeitz@usz.ch

PD Dr. med. Daniel Pohl, Oberarzt

Klinik für Gastroenterologie und Hepatologie

UniversitätsSpital Zürich

Rämistrasse 100

8091 Zürich

E-mail: daniel.pohl@usz.ch

**Microbiologists**:

**Principal Investigator**:

Prof. Dr. Wolf-Dietrich Hardt

Institut für Mikrobiologie

ETH Zürich

Vladimir-Prelog-Weg 4

CH-8093 Zurich, Switzerland

E-mail: wolf-dietrich.hardt@micro.biol.ethz.ch

**Additional investigators:**

Dr. Lisa Maier

Institut für Mikrobiologie

ETH Zürich

Vladimir-Prelog-Weg 4

CH-8093 Zurich, Switzerland

E-mail: lisa.maier@micro.biol.ethz.ch

Substitutes (all Institut für Mikrobiologie):

Dr. Emma Slack emma.slack@micro.biol.ethz.ch

Manja Barthel barthelm@micro.biol.ethz.ch

Sandra Wotzka [wotzkas@ethz.ch](mailto:wotzkas@ethz.ch)

Kathrin Moor kathrin.moor@micro.biol.ethz.ch

Markus Kreuzer kreuzmar@ethz.ch

Dr. Bidong Nguyen [nguyenb@ethz.ch](mailto:nguyenb@ethz.ch)

Dr. Markus Schlumberger schlumar@ethz.ch

Ilka Riedel iriedel@ethz.ch

Institut für Mikrobiologie

ETH Zürich

Vladimir-Prelog-Weg 4

CH-8093 Zurich, Switzerland

**Signatures, protocol permission and responsible individuals:**

**Sponsor:**

Prof. Dr. med. Dr. phil. Gerhard Rogler

Klinik für Gastroenterologie und Hepatologie

UniversitätsSpital Zürich

Rämistrasse 100

8091 Zürich

E-mail: Gerhard.Rogler@usz.ch

Date, Signature…………………………………………………………………………..

**Principal Investigator:**

PD Dr. med. Benjamin Misselwitz

Klinik für Gastroenterologie und Hepatologie

UniversitätsSpital Zürich

Rämistrasse 100

8091 Zürich

E-mail: benjamin.misselwitz@usz.ch

Date, Signature…………………………………………………………………………..

**Microbiologist:**

Prof. Dr. Wolf-Dietrich Hardt

Institut für Mikrobiologie

ETH Zürich

Vladimir-Prelog-Weg 4

CH-8093 Zurich, Switzerland

E-mail: wolf-dietrich.hardt@micro.biol.ethz.ch

Date, Signature…………………………………………………………………………..

1. STUDY SYNOPSIS

| Study Title: | Elucidating the influence of lactulose intake on the gut microbiota composition. |
| --- | --- |
| Protocol Version and Date: | Protocol ID: KEK-ZH-Nr.  Version: 1.0  Date: 16.6.2014 |
| Methodology: | This is a clinical study for investigating the influence of lactulose (as used for the lactulose breath test) on the composition and metabolic activities of the intestinal microbiota. Healthy volunteers will donate fecal samples and urine before (24 h) and after (1-2d, 14d) a routine lactulose breath test. Samples will be analyzed for their microbiota composition and key metabolites. |
| Study Duration: | Data acquisition for the study will be performed from October 2014 to March 2016. Total study duration for each participant is 4 hours maximum (including preparation time) at the study center for the breath test plus collection of fecal samples and urine. |
| Study Center: | Division of Gastroenterology and Hepatology  University of Zurich  Rämistrasse 100  8091 Zürich |
| Objective(s): | Primary objective:   - Influence of lactulose intake on gut microbiota composition   Secondary objectives:   - Influence of lactulose intake on metabolic profiles within the fecal ecosystem and the host (urine) |
| Number of Subjects: | 40 healthy volunteers |
| Diagnosis and Main Inclusion Criteria: | - Subjects free of abdominal complaints (=healthy). - Written informed consent - Working at ETH Zurich/University of Zurich, experience in handling of liquid nitrogen on biosafety level 2 |
| Main Exclusion Criteria: | - Age under 18 - Previous history of gastrointestinal disease or surgery (excludes appendectomy, hernia repair and anorectal disorders) - Known diabetes mellitus, scleroderma, neurological impairment or other major current disease - Subjects unable to stop medication that alters gut flora: proton pump inhibitors, laxatives and antibiotics at least 4 weeks before study entry. - Pregnancy beyond week 12 (no pregnancy test will be performed) - Involvement in any other clinical trial during the course of this trial, nor within a period of 14 days prior to its beginning or 14 days after its completion |
| Study Schedule: | October 2014 – March 2016: Recruitment, data collection and analysis. |
| Statistical Methodology: | Acquired H_2_-measurements and metabolic profiles will be analyzed according to standard algorithms. Several algorithms are available (see below). Microbial gut communities will be analyzed by 16S rRNA sequencing and compared using hpc-clust (see below). |
| Statement: | This study will be conducted in compliance with the protocol, the current version of the Declaration of Helsinki („59th WMA General Assembly“, Seoul, October 2008), and ICH-GCP as well as all national legal and regulatory requirements. |

- 1. Study Flow chart

Informed consent will be obtained during a separate visit. The study will be finished within 15 days for each individual, with one main study day. The following basic steps will be performed:

- Informed consent will be obtained during the first study visit. At that time,basic medical history and gastrointestinal complaints will be addressed, dates for the lactulose breath test will be agreed on

- One to three days before the lactulose or sham test the volunteer collects a fecal and a urine sample using the restrooms of his/her workplace at the Institute of Microbiology, ETH Zurich and directly snap-freeze it using liquid nitrogen. The samples will be stored by the proband at -80°C (at biosafety level 2).

- a lactulose breath test or a sham test will be carried out

- Within the next two days, the proband will collect a fecal and urine sample at his/her workplace, snap-freeze it and store it at -80°C.

- 14 days after the lactulose breath test, the volunteer will take additional fecal and urine samples following the same procedure.

- Finally, all stool and urine samples will be assembled at ETH for further processing.

- 1. List of abbreviations

AE Adverse Event

BMI Body Mass Index

CRF Case Report Form

GCP Good Clinical Practice

GI Gastrointestinal

LHT Law on human research

HMG Heilmittelgesetz

ICH International Conference on Harmonisation

IEC Independent Ethics Committee

ClinO Clinical Trial Ordinance

i.g. Intragastric

h hour

g gramm

Art.nr article number

BL2 Biosafety Level 2

1. INTRODUCTION
   1. Background

Humans and other animals coexist with trillions of microbes on their body surfaces, collectively referred to as microbiota. One of the key benefits these microbes confer to their host is colonization resistance (CR), i.e. protection against invasion and infection by pathogens. It is conceivable that during gut colonization, the pathogen has to compete with the resident intestinal microbiota for nutrients and binding sites (nutrient-niche hypothesis) [1, 2]. Yet, the mechanistic details underlying the protective effect by the microbiota have to be elucidated.

During murine infections with *Salmonella* Typhimurium (*S*. Tm), colonization resistance can be disrupted by disturbing the microbiota via antibiotic treatment [2-4]. Likewise, according to the nutrient-niche hypothesis, providing the ecosystem with an additional nutrient should also open new niches, which might reduce colonization resistance for certain pathogens. To test this hypothesis, we fed mice with the disaccharide lactulose, which cannot be absorbed during the passage through the host's small intestine [5, 6]. Therefore, it enriches the nutrient pool available to the gut microbes in the large intestine. *S*. Tm cannot grow on lactulose itself but might profit directly or indirectly from the sugar monomers galactose and fructose which are released upon degradation of lactulose by certain members of the gut microbiota [7, 8]. Lactulose-feeding of CR-proficient mice resulted in a significantly higher colonization and inflammatory responses by *S*. Tm than in untreated mice. Therefore, the increased intake of lactulose seems to rise the infection risk for this enteropathogen.

These findings have direct medical implications, as lactulose is used as a food ingredient (named galactofructose), for the treatment of constipation and hepatic encephalopathy, and in the lactulose breath test to diagnose small intestinal bacterial overgrowth [9].

- 1. Rationale for current study

In this study, we aim at monitoring the effects of lactulose intake on human patients undergoing a lactulose breath test. In particular, we would like to monitor shifts in the microbial metabolic landscape and microbial composition (e. g. enterobacterial blooms) as a direct consequence of lactulose uptake.

- 1. Aim and significance of the project

The results gained from these analyses will help us to elucidate the nutritional effects of non-digestible polysaccharides on the microbiota composition and its metabolites. Furthermore, the insights might challenge the concept of prebiotics, as they might not be exclusively beneficial but also be unsafe by opening new niches for pathogenic invaders.

- 1. Categorization of the study

This study is classified as risk category A, as it is a health-related study intervention with only minimal risk and burdens and as the lactulose breath test is performed routinely as a diagnostic method.

1. STUDY DESIGN

This is a clinical study in healthy volunteers aiming to investigate the influence on lactulose intake on the gut microbiota composition and on microbiota-dependent metabolites.

- 1. Primary and Secondary objectives

**Primary objective:**

- Change in microbial community composition upon lactulose intake

**Secondary objectives:**

- Changes in metabolic profiles upon lactulose intake within the gut microbial community (measured in fecal samples) and its impact for the host metabolism (measured in urine samples).
- Inflluence of basic epidemiological parameters (age, gender, country of origin, exercise, smoking) on composition of gut microbiota.
- Influence of microbiota on hydrogen concentration in lactulose breath test
  1. Study procedures, methods outcomes

For this study we will recruit healthy volunteers from ETH Zurich/University of Zurich. Only individuals with training and experience handling liquid nitrogen and -80°C freezers (BL2) will be included in this study. The volunteers will use goggles, gloves and will wear lab coats to snap-freeze the fecal samples. For encoding, cryo-tubes will be labeled with the study number and the day only and the name and personal information will only be known to the physician recruiting the proband for the study. .

The first sample of stool and urine will be collected by the proband one to three days before the lactulose breath test (date will be indicated on the tube). Subsequently, the volunteers will undergo a lactulose breath test or a sham test. Probands will be randomized using a sealed envelope method to receive either 50g lactulose or 50g sucrose in 200 ml water. The study design will be double blinded and neither the study nurse nor the proband will know about group assignment. After ingestion of lactulose/sucrose, hydrogen concentrations in the breath of the individuals will be monitored (baseline level, 30 min, 60 min and 120 min after lactulose/sucrose intake).

Within the next two days, the proband will collect a fecal and urine samples during his or her working hours as described above (date will be indicated on the tube). 14 days after the breath test, a third fecal and a third urine sample will be collected.

Before the study, there will be one study visit to inform the probands and to obtain informed consent and address basic medical history and gastrointestinal complaints. This is done by one of the MDs in a seminar room at the Institute of Microbiology, ETH Zürich. Specifically, probands will be asked about loss of appetite, nausea, early satiety, sense of abdominal fullness, vomiting, abdominal pain, dysphagia, heartburn, reflux, sweating/ shivering after meals and diarrhea. In addition, probands will be asked about their current medication (proton pump inhibitors, antibiotic therapy, laxative drugs within the last four weeks) previous abdominal surgery, additional chronic diseases, pregnancy, and current or past (≤2 weeks) participation in addition clinical trials (questionnaire Appendix), eating habits (vegetarian, vegan), ethnicity, smokers/non-smokers, the amount of regular physical exercise, stool frequency/consistency (Bristol Stool Scale), weight and height.

The study will be finished within approximately 16 days for each individual. The study includes one examination of approximately 4 hours, the remaining parts of the studies (collection of feces and urine and proper storage of these samples) will be done at the working place of the proband (ETH Hoenggerberg, Zurich).

The anticipated study duration for is from October 2014 to March 2016. Total study duration for each participant is 4 hours maximum (including preparation time) at the study center for the breath test plus collection of fecal samples and urine.

- 1. Method of Randomization and Blinding

To randomly assign our probands to lactulose/sucrose groups, we will apply a sealed envelope technique. Lactulose and sucrose (supermarket) will be filled in tubes labeled with numbers indicated within the envelope. That way, neither the proband nor the study nurse/PI will know the group assignment. Randomization will be ensured using the following program:

<http://www.graphpad.com/quickcalcs/randomize1.cfm>

- 1. Method of encryption

To ensure encryption of samples , cryotubes with fecal and urine samples will be pre-labeled with the study number and the respective date respectively to the breath test. The key for encryption will be stored at University Hospital Zurich. Therefore, only the principal medical investigator, but not the microbiologists will be able to decode the study number and assign this identifier to an individual.

- 1. Unblinding Procedures (Code break)

An Emergency Code Break will be available to the investigator. This Code Break should be opened only in emergency situations when the identity of the investigational intervention must be known by the investigator in order to provide appropriate medical treatment.

1. Recruitment / Participant Entry / Discontinuation
   1. Pre Registration Evaluations

Volunteers will be recruited via public advertisement on notice boards at ETH Hoenggerberg Zurich, as well as by an Email to all members of the Institute of Microbiology and other departments at ETH Hoenggerberg. Exemplary advertisements/Email for potential volunteers are shown in the appendices. Potential volunteers can contact Dr. Lisa Maier, who will give a short summary of the aim and benefits of the study and will check whether the potential volunteers can be included in the study (inclusion criteria/exclusion criteria). No individual-related data will be registered during this telephone interview. (See document 4b-Rekrutierung_Telefonfragebogen).

Interested subject will receive study information by email. Medical history and current treatment will be extracted through a standardized interview before the study after informed consent has been obtained. This is done by one of the MDs in a seminar room at the Institute of Microbiology, ETH Zürich.

- 1. Inclusion criteria

• Subjects free of abdominal complaints or symptoms

• Written informed consent

• Working at ETH Zurich/University of Zurich and trained and experienced in handling liquid nitrogen/-80°C freezers at biosafety level 2.

• Age: 18 to 85 years

- 1. Exclusion criteria
- Previous history of gastrointestinal disease or surgery (excludes appendectomy, hernia repair and anorectal disorders)
- Known diabetes mellitus, scleroderma, neurological impairment or other major current disease
- Subjects with antibiotic therapy, proton pump inhibitors or laxatives within the last four weeks.
- Pregnancy beyond week 12. The lactulose breath test is safe also during pregnancy; however, pregnancy might alter the human microbiota and there is a slight risk that advanced pregnancy would be a confounder in our study. No pregnancy test will be performed.
- Involvement in any other clinical trial as proband during the course of this trial or within a period of 14 days prior to its beginning
  1. Criteria for discontinuation

Subjects may be discontinued from study treatment and assessments at any time, at the discretion of the Principle Investigators. Specific reasons for discontinuing a subject from this study are:

• Withdrawal of informed consent

• Protocol non-compliance (protocol deviation)

- - 1. Voluntary discontinuation by a subject

Subjects are free to discontinue their participation in the study at any time. Subjects who discontinue from the study should always be asked about the reason(s) for their discontinuation and about the presence of any adverse events. Adverse events should be followed up. Data of these probands will be analyzed. However, data of probands will be anonymized and all contact data and identifying information will be deleted from the subject identification log..

1. Efficacy variables

6.1. Demographic measurements

Parameters for describing proband characteristics in this study are:

- Body weight [kg], body height [cm], BMI [kg/m^2^]

- Sex [male/female]

- Age [year]

- Medical history

- Medication use

- eating habits/diet restraints (vegan, vegetarian,…)

- ethnicity

- smokers/ non-smokers

- level of regular physical training

6.2. Parameters for assessing protocol compliance

Parameters for assessing protocol compliance in this study are (checked by study nurse):

- Refrain from substances that might alter gut microbiota (including proton pump inhibitors, laxatives and antibiotics) unless medically necessary

- Collection of the required samples (3 fecal samples, 3 urine samples)

- Completion of measurement of hydrogen content of exhaled air after lactulose intake (lactulose breath test).

1. SAFETY of conducted procedures
   1. Fecal and urine sample collection

Volunteers will be provided with individually numbered cryotubes and will be asked to collect fecal and urine samples using the restrooms at the Institute of Microbiology, ETH Zurich. After sample collection, the proband snap-freezes the samples in liquid nitrogen and transfers the samples to a -80°C freezer (Biosafety Level 2). Liquid nitrogen and -80° freezers can be handled very safely with some basic training and compliance with few basic precautions. During recruitment we will ensure that all probands are trained in handling liquid nitrogen and 80°C freezers at biosafety level 2 (this is the case for near all scientific members at ETH). All probands will wear suitable protection (gloves and googles). As handling liquid nitrogen is part of their regular working routine, this step will not impose an additional risk to our volunteers. Furthermore, handling of stool or urine from other individual carries a small risk for infections. However, this is not the case if an individual handles his or her own stool or urine and autoinfection is not a concern.

7.2. Lactulose breath test

The lactulose breath test is a non-invasive, routine diagnostic test. Breath samples are obtained with hand-held breath test devices before and after intake of 50 g lactulose/sucrose. Exhaled hydrogen concentrations can be measured in parts per million (ppm). The technology is perfectly safe. Minor adverse events include diarrhea, bloating and mild abdominal pain. Sever adverse events in healthy individuals are unknown.

- 1. Procedures for discontinuation

If a subject discontinues participation one of the investigators will immediately be called. If a subject is withdrawn, the subject will have contact to the principal investigator (if possible) or one of the additional investigators. Adverse events will be followed up. Data of these subjects will be analyzed as censored at the time of withdrawal. If no analysis of the microbial composition/metabolites within the samples is possible due to limited data over time, measurements will be regarded as a drop out.

1. STATISTICS AND DATA ANALYSIS
   1. Analysis of fecal and urine samples

**Microbial composition:**

- Microbial DNA will be extracted from fecal samples and analysed as described previously [10] by member of the Hardt lab, Institute of Microbiology, ETH Zurich. 16S rRNA sequencing will be performed in collaboration with the Functional Genomics Center Zurich and sequencing reads will be evaluated according to established protocols [10] by the von Mering lab, Swiss Institute of Bioinformatics, University of Zurich by HPC-CLUST. Additional genetic, microbiological or immunological analyses are also possible.

**Metabolite composition:**

- Monitoring compositional metabolic changes requires the identification and quantification of large portions of metabolites that constitutes these metabolomes. Such global profiling of samples can be achieved by untargeted metabolomics on the basis of mass spectrometry [21]. After metabolite extraction (Hardt lab), samples will be analysed by the Sauer lab, Institute of Molecular Systems Biology, ETH Zurich. Additional metabolic analyses are also possible.
  1. Lactulose breath test

The lactulose breath test will be evaluated according the standard procedures using established algorithms. Hydrogen concentrations on both groups (lactulose vs. sucrose intake) will be compared using the Mann-Whitney U test.

- 1. Significance level

Significance levels will be determined as described in [12].

- 1. Dropouts

Dropouts will be replaced until 40 (20 for each group) complete sets of samples including the results for the lactulose breath test are available.

1. Study related regulatory issues
   1. Recruitment and Informed Consent

Prior to study enrolment, written informed consent must be obtained from each subject. It is the responsibility of the investigator to obtain this written informed consent after adequate explanation to the subject of the aims, methods, source of funding, the anticipated benefits and potential risks of the study and the discomfort it may entail. Written informed consent should be obtained prior to screening, with the understanding that consent may be withdrawn at any time without prejudice. Two copies of the informed consent are signed: one is given to the subject and one is retained in the Investigator Site File on site.

The right of the participant to refuse to participate without giving reasons must be respected. All participants are free to withdraw at any time from the protocol without giving reasons and without prejudicing possible future treatments at our facility. There is no state of dependence between the proband and the investigators.

- 1. Confidentiality

The Principal Investigator will preserve the confidentiality of participants taking part in the study and is registered under the Data Protection Act.

- 1. Funding

Prof. W.-D. Hardt, Institute of Microbiology, ETH Zurich and "Klinik für Gastroenterologie and Hepatologie", Universitätspital Zürich, are funding this study.

- 1. Participant compensation

For completing this study subjects will receive a compensation of 200.- CHF for the discomfort they may experience while participating in the trial (mainly loss of time, collection of fecal samples might cause discomfort, lactulose breath test). For completing only a part of the study, no compensation will be granted. Volunteers will be reimbursed after handing over the last set of samples (14 day after the lactulose breath test).

- 1. Preventive measures

Participants will be asked about their experience in handling liquid nitrogen and -80°C freezer at biosafety level 2. If they feel uncomfortable in snap-freezing their samples or storing them in a -80°C freezer, they will be excluded.

1. Duties of the principle investigator
   1. GCP

The study will be conducted in accordance with principles enunciated in the current Declaration of Helsinki („59th WMA General Assembly“, Seoul, October 2008), the guidelines of Good Clinical Practice (GCP) issued by ICH, and Swiss regulatory authority’s requirements. The Principal Investigator will require a copy of the Ethic’s approval letter before accepting participants into the study.

- - 1. Criteria for premature termination of the trial

The Principle Investigator reserves the right to discontinue the trial at any time. In terminating the trial, the investigator will assure that adequate consideration is given to the protection of the interests of all subjects. The Principle Investigator must notify the Ethics Committee of the premature termination.

- - 1. Publication policy

The results of this study will be presented as scientific papers in an adequate journal. This trial will be registered in a public clinical trial database (anticipated: SNCTP and ICTRP).

- 1. Reporting Procedures

All adverse events will be reported. Depending on the nature of the event the reporting procedures below should be followed. Any questions concerning adverse event reporting will be directed to the Principal Investigator in the first instance. The Principle Investigator will report severe adverse reactions, changes (to the protocol), intermediate and final reports to the ethic commission.

- - 1. Definitions

**AE**

An Adverse Event (AE) is any untoward medical occurrence in a patient or clinical investigation subject administered a study product and which does not necessarily have a causal relationship with this treatment (ICH-GCP definition).

**SAE**

A Serious Adverse Event (SAE) is any untoward medical occurrence or effect that at any dose:

- results in death

- is life-threatening (at the time of the event)

- requires hospitalization or prolongation of existing subjects’ hospitalization

- results in persistent or significant disability or incapacity

- results in a congenital anomaly or birth defect

- - 1. (S)AE recording

Any (S)AE as reported spontaneously by the subject or observed by the investigator or staff, is recorded on the AE form during the course of the study. The investigator must ensure that this information, including onset, duration and nature of event, severity, and action taken, is captured. SAEs must additionally be recorded on the SAE report form.

The severity of any (S)AE is scored as follows:

**Mild:** transient or mild discomfort; no medical intervention/therapy required

**Moderate:** mild to moderate limitation in activity; some assistance may be needed; and/or minimal medical intervention/therapy required

**Severe:** marked limitation in activity; some assistance usually required; and/or significant medical intervention/therapy/hospitalization required

The relationship of the (S)AE to the study product is assessed as being “not related / unlikely / possibly / probably / definitely related”.

- - 1. SAE reporting by the investigator

The investigator must report the SAE to the principal investigator as soon as possible. The investigator must complete the SAE report within 48 hours (working days) after first notice. The follow-up report should be completed as soon as possible and handed over the report to the principal investigator.

- - 1. SAE review and reporting by the Sponsor

The principle investigator must review all reported SAEs. The principle investigator must report all deadly SAEs and all life threatening SAEs causally related to the study immediately (within 7 days) to the accredited Ethics Committee that approved the protocol. Other SAEs causally related to the study must be reported within 15 days. SAEs are reported annually as line listings or according to the requirements of the Ethics Committee.

- - 1. Follow-up of (S)AEs

All (S)AEs are followed-up by the investigator until they have abated, or until a stable situation has been reached. Depending on the event, follow-up may require additional tests or medical procedures as indicated, and/ or referral to the general physician or a medical specialist.

- 1. Insurance

Insurance is covered by the general liability insurance of the University hospital Zurich.

Any damage developed in relation to study participation is covered by this insurance. So as not to forfeit their insurance cover, the participants themselves must strictly follow the instructions of the study personell. Participants must not be involved in any other medical treatment without permission of the principal investigator (emergency excluded). Medical emergency treatment must be reported immediately to the investigator. The investigator must also be informed instantly, in the event of health problems or other damages during or after the course of study treatment.

The investigator will allow delegates of the insurance company to have access to the source data/documents as necessary to clarify a case of damage related to study participation. All involved parties will keep the patient data strictly confidential.

1. Ethical considerations
   1. Ethics committee

This protocol and any accompanying material provided to the subjects, such as information and informed consent sheets, are submitted to the applicable Ethics Committee by the investigator according to local legislation. Approval from the Ethics Committee must be obtained before starting the study, and should be documented in a letter to the investigator specifying the date on which the Ethics Committee met and granted the approval, the composition of the Ethics Committee, and version and date of all submitted documents. The investigator must submit a status report at least annually to the Ethics Committee that approved the protocol.

- 1. Confidentiality of study data

Data generation, transmission, archiving and analysis of personal data within this project, strictly follows Swiss legal requirements for data protection. The Principal Investigator will preserve the confidentiality of probands whose data are analyzed and is registered under the Data Protection Act. The Sponsor and the Principal investigator are responsible for the protection of personal information.

For the purpose of this study, probands data will be encrypted. Only the persons mentioned as clinical investigators and additional investigators of this study will see or receive non-anonymized data (point 1: general information). For the subsequent scientific analysis only encrypted data will be used and under no circumstances will additional researches learn about the name of the proband or other information which would make identification possible.

For encryption each proband will have a unique study number. This number will be used throughout the study for entry into an electronic form and subsequent analyses. Information facilitating identification of individual probands will not be entered. The key for encryption will be stored at the Functional Lab at USZ. Only the researchers mentioned as clinical investigators and additional investigators of the study will be able to view the key for encryption and will have the option to match the study number to an individual proband.

Encrypted proband information including the CRF and the data base will be stored at the department of microbiology at ETH Zurich. For additional protection, the data base will be password protected. The data will be stored at the servers of the ETH for at least 10 years after the publication of the last data of this analysis.

- 1. Benefits and risks assessment
     1. Safety of sample collection and storage

Probands will be asked about their confidence in handling liquid nitrogen to snap-freeze fecal and fecal samples. As all proband are employed at ETH Zurich/University of Zurich and trained and experienced in liquid nitrogen handling and -80 degree freezers (biosafety level 2), this procedure will not impose safety risks complementary to the risk exposure of a usual working routine of our proband.

- - 1. Safety of lactulose breath test

The lactulose breath test is a non-invasive diagnostic test with is routinely used at the Klinik für Hepatologie und Gastroenterologie, Unispital Zürich. The test is almost perfectly safe. Minor adverse events include discomfort, diarrhea, bloating and mild abdominal pain. Sever adverse events in healthy individuals are unknown.

- - 1. For Women

Female volunteers are not allowed to take part in the study, if a pregnancy beyond week 12 is known. The test is safe during pregnancy and for children; however, the composition of the gastrointestinal microbiota might be affected. All women will be asked about a likely pregnancy, however, since there is no risk for the woman or the unborn child, no pregnancy test will be performed.

- 1. Benefits of the current study

There will no benefit for the participants of this study. As a result of the study, the effect of lactulose intake on the gut microbiota composition will tell us, whether increased consumption of non-digestible polysaccharides might destabilize the microbial community. This in turn might increase the infection risk with enteropathogens. This benefit together with the lack of risk and only minimal inconvenience for the test subjects argue in our point of view strongly for this study.

- 1. Compensation for subjects

The study subjects will receive a financial compensation (200,- CHF) for participation in the study.

1. Quality control

The day-to-day management and coordination of the study will be coordinated through PD Dr. med. B. Misselwitz, Prof. Dr. med. Mark Fox, Dr. med. D. Pohl and Dr. med. I. Ulmer. There won't be any further monitoring as there is basically no risks for the probands and only one visit in the hospital for the lactulose breath test.

- 1. Audits

The study may be subject to inspection and audit by regulatory bodies to ensure adherence to GCP, national law, and regulatory requirements.

- 1. Source data and data encryption

Source data include the medical history of the proband and questionnaires filled out by the proband before the examination (compare respective files). Source data will subsequently be transferred to the CRF. The investigators will use paper or electronic case report forms (CRF), one for each enrolled study participant, to be filled in with all relevant data pertaining to the participant during the study. All participants who either entered the study or were considered not-eligible or were eligible but not enrolled into the study additionally have to be documented on a screening log. The investigator will document the participation of each study participant on the Enrolment Log. Each subject will receive a screening number. Encoding is ensured by listing only the study number on the CRF. Only the CRF will be used for subsequent scientific evaluation of the study. Data from the lactulose breath test will be transferred to an electronic form.

- 1. Data handling

Subject data will be collected during screening on an anonymized CRF and will be entered in an electronic form. During and at the end of the project period data will be transferred into the electronic form. After the end of data collection the electronic form will be frozen.

- - 1. Documentation and storage

The investigator will create an Investigator Site File and is responsible to keep this Investigator Site File updated and available for review by a study monitor. All paper documents will be stored at the investigator site (Klinik für Gastroenterologie and Hepatologie, UniverstätsSpital Zürich) in room A-West 23. All documents pertaining to the conduct of the study must be kept by the investigator for a period of 10 years.

- 1. Sample storage and further analysis

After finishing this study, fecal and urine samples will be stored in a -80°C freezer at the department of microbiology, ETH Zurich (Hönggerberg). Depending on the outcome of this study additional analysis might be of interest (e.g. genome sequencing of some isolates of the gut microbiota). Additional microbiological, genetic, immunological and biochemical analyses might follow.

1. Suitability of the place of inspection
   1. Team

The team of MDs at the UniversitätsSpital are routinely using the lactulose breath test in diagnostics. They participated and conducted several clinical trials so far and all are GCP-trained. The team of scientist at the Institute of Microbiology, ETH Zürich have all the experimental methods and bioinformatics running to analyze the samples collected during this study.

- 1. Infrastructure

The Institute of Microbiology in cooperation with the Functional Genomics Center, Prof. Christian von Mering and Prof. Uwe Sauer can provide all the equipment necessary to analyze the samples collected in this study. The required equipment for the lactulose breath test is available at UniversitätsSpital Zürich.

1. References

1. Stecher, B., D. Berry, and A. Loy, Colonization resistance and microbial ecophysiology: using gnotobiotic mouse models and single-cell technology to explore the intestinal jungle. FEMS Microbiol Rev, 2013. 37(5): p. 793-829.

2. Stecher, B. and W.D. Hardt, Mechanisms controlling pathogen colonization of the gut. Curr Opin Microbiol, 2011. 14(1): p. 82-91.

3. Barthel, M., et al., Pretreatment of mice with streptomycin provides a Salmonella enterica serovar Typhimurium colitis model that allows analysis of both pathogen and host. Infect Immun, 2003. 71(5): p. 2839-58.

4. Bohnhoff, M., B.L. Drake, and C.P. Miller, Effect of streptomycin on susceptibility of intestinal tract to experimental Salmonella infection. Proc Soc Exp Biol Med, 1954. 86(1): p. 132-7.

5. Dahlqvist, A., and Joyce D. Gryboski, Inability of the human small-intestinal lactase to hydrolyze lactulose. Biochimica et Biophysica Acta (BBA)-Enzymology and Biological Oxidation 110.3 (1965): 635-636, 1965.

6. Hoffmann K., et al., Studies on the mechanism of action of lactulose (beta-galactoside action of lactulose (beta-galactosido-fructose) in the intestine. . Klinische Wochenschrift [1964, 42:126-130] 1964.

7. El Kaoutari, A., et al., The abundance and variety of carbohydrate-active enzymes in the human gut microbiota. Nat Rev Microbiol, 2013. 11(7): p. 497-504.

8. Flint, H.J., et al., Polysaccharide utilization by gut bacteria: potential for new insights from genomic analysis. Nat Rev Microbiol, 2008. 6(2): p. 121-31.

9. Schuster-Wolff-Buhring, R., L. Fischer, and J. Hinrichs, Production and physiological action of the disaccharide lactulose. International Dairy Journal, 2010. 20(11): p. 731-741.

10. Maier, L., et al., Microbiota-derived hydrogen fuels Salmonella Typhimurium invasion of the gut ecosystem, 2013 Cell Host & Microbe, 2013 Dec 11;14(6):641-51.

11. Patti, G. J., et al., Innovation: Metabolomics: The apogee of the omics triology, Nat Rev Mol Cell Biol 13, 263 (April, 2012a)

12. Matias Rodrigues, J. F., HPC-CLUST: Distributed hierarchical clustering for very large sets of nucleotide sequences, Bioinformatics. 2014 Jan 15;30(2):287-8

13. Eisenmann A., et al., Implementation and interpretation of hydrogen breath tests, J. Breath Res. 2 (2008) 046002 (9pp)
