# Supplementary material for: Microbiota stability in healthy individuals after single-dose lactulose challenge—A randomized controlled study
Source: PLoS One. 2018 Oct 25;13(10):e0206214. doi: 10.1371/journal.pone.0206214 (PMC6201941; doi:10.1371/journal.pone.0206214)

A

Order	Family	Genus	Species
Enterobacteriales	Enterobacteriaceae	*Escherichia-Shigella*	*Escherichia coli*
Enterobacteriales	Enterobacteriaceae	Unknown	Unknown
Enterobacteriales	Enterobacteriaceae	*Hafnia-Obesumbacterium*	Unknown
Enterobacteriales	Enterobacteriaceae	*Raoultella*	Unknown
Enterobacteriales	Enterobacteriaceae	*Proteus*	*Proteus*
Enterobacteriales	Enterobacteriaceae	*Yersinia*	*Yersinia*

B

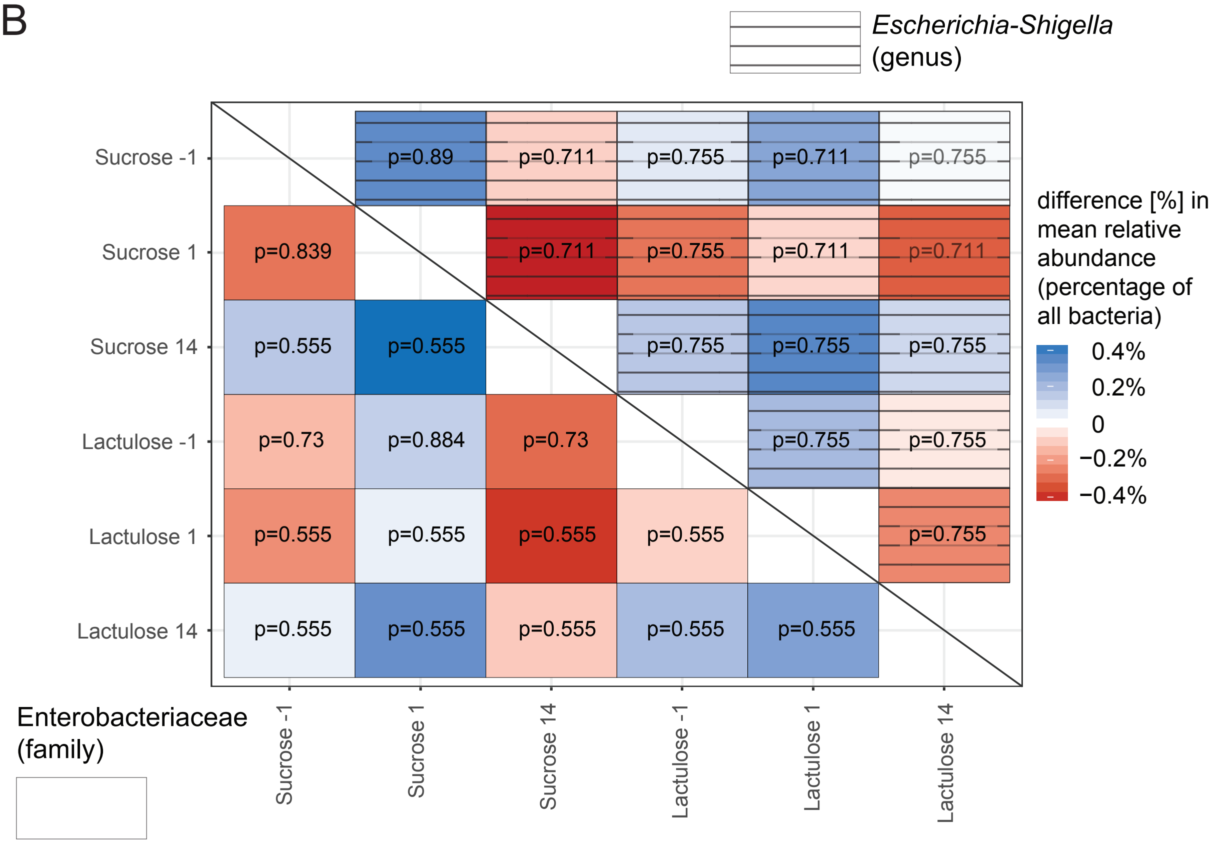

Supplement: S2 Fig — (A) Taxonomic annotation of five OTUs of the family of Enterobacteriaceae are shown, one of which belongs to the genus Escherichia/ Shigella, which had a 100% sequence match to Escherichia coli. (B) The relative abundance of members of the genus Escherichia-Shigella (striped, upper right part) and the family Enterobacteriaceae (lower left part) show no significant (p<0.05) differences according to treatment (lactulose vs. sucrose) or between time points (paired Wilxocon test, with FDR correction). The numbers in the boxes are FDR corrected p-values for the group comparisons. The color key indicates the difference [%] in mean relative abundance (percentage of all bacteria) between two groups ri–cj, where ri represents the mean relative abundance of the group identified by the row label and cj the mean relative abundance of the group identified by the column label. Difference in abundance is expressed as mean difference of the percentages. (PDF) [file pone.0206214.s004.pdf]
