# Supplementary material for: Microbiota stability in healthy individuals after single-dose lactulose challenge—A randomized controlled study
Source: PLoS One. 2018 Oct 25;13(10):e0206214. doi: 10.1371/journal.pone.0206214 (PMC6201941; doi:10.1371/journal.pone.0206214)
Supplement: S1 Table. Detailed quantification of symptoms by individual participants — (DOCX) [file pone.0206214.s005.docx]

|  |  | nausea | | | | | bloating | | | | | diarrhea | | | | | borborygmi | | | | | abdominal pain | | | | |
| --- | --- | --- | --- | --- | --- | --- | --- | --- | --- | --- | --- | --- | --- | --- | --- | --- | --- | --- | --- | --- | --- | --- | --- | --- | --- | --- |
|  | time (min) | 1 | 2 | 3 | 4 | 5 | 1 | 2 | 3 | 4 | 5 | 1 | 2 | 3 | 4 | 5 | 1 | 2 | 3 | 4 | 5 | 1 | 2 | 3 | 4 | 5 |
| sucrose | 0 | 15 |  |  |  |  | 15 |  |  |  |  | 15 |  |  |  |  | 14 | 1 |  |  |  | 15 |  |  |  |  |
|  | 15 | 14 | 1 |  |  |  | 13 | 2 |  |  |  | 15 |  |  |  |  | 15 |  |  |  |  | 15 |  |  |  |  |
|  | 30 | 14 | 1 |  |  |  | 13 | 2 |  |  |  | 15 |  |  |  |  | 15 |  |  |  |  | 15 |  |  |  |  |
|  | 60 | 15 |  |  |  |  | 12 | 3 |  |  |  | 15 |  |  |  |  | 15 |  |  |  |  | 14 | 1 |  |  |  |
|  | 90 | 13 | 1 | 1 |  |  | 13 | 2 |  |  |  | 15 |  |  |  |  | 15 |  |  |  |  | 15 |  |  |  |  |
|  | 120 | 14 | 1 |  |  |  | 15 |  |  |  |  | 15 |  |  |  |  | 14 | 1 |  |  |  | 15 |  |  |  |  |
|  | 180 | 15 |  |  |  |  | 15 |  |  |  |  | 15 |  |  |  |  | 15 |  |  |  |  | 15 |  |  |  |  |
| lactulose | 0 | 16 | 1 |  |  |  | 16 | 1 |  |  |  | 17 |  |  |  |  | 17 |  |  |  |  | 17 |  |  |  |  |
|  | 15 | 16 | 1 |  |  |  | 14 | 3 |  |  |  | 17 |  |  |  |  | 16 | 1 |  |  |  | 16 | 1 |  |  |  |
|  | 30 | 16 | 1 |  |  |  | 12 | 5 |  |  |  | 17 |  |  |  |  | 15 | 2 |  |  |  | 16 | 1 |  |  |  |
|  | 60 | 15 | 2 |  |  |  | 7 | 8 | 2 |  |  | 15 | 1 |  | 1 |  | 10 | 6 | 1 |  |  | 14 | 3 |  |  |  |
|  | 90 | 14 | 2 | 1 |  |  | 4 | 9 | 2 | 2 |  | 10 | 1 | 5 |  | 1 | 8 | 5 | 3 | 1 |  | 10 | 4 | 2 | 1 |  |
|  | 120 | 15 | 2 |  |  |  | 4 | 9 | 4 |  |  | 10 | 3 | 3 | 1 |  | 4 | 9 | 3 | 1 |  | 9 | 5 | 1 | 2 |  |
|  | 180 | 15 | 2 |  |  |  | 3 | 8 | 5 | 1 |  | 9 | 4 | 2 | 1 | 1 | 3 | 10 | 3 | 1 |  | 9 | 6 |  | 2 |  |
