# Supplementary material for: Microbiota stability in healthy individuals after single-dose lactulose challenge—A randomized controlled study
Source: PLoS One. 2018 Oct 25;13(10):e0206214. doi: 10.1371/journal.pone.0206214 (PMC6201941; doi:10.1371/journal.pone.0206214)
Supplement: S2 Table — Dark grey indicates resistance against the respective antibiotics, medium grey indicates intermediate resistance against the tested antibiotic (this depends on the antibiotic) and light grey means susceptibility to the antibiotic. (DOCX) [file pone.0206214.s006.docx]

|  |  | Diameter of zone of inhibition (mm) | | | | | | |
| --- | --- | --- | --- | --- | --- | --- | --- | --- |
| Sample | Name (By 16S rRNA) | Cefepime | Gentamicin | Ciprofloxacin | Imipenem | Sulfamethoxazole + Trimethoprim | Ampicillin | Kanamycin |
| 1.2 | Escherichia | 36 | 26 | 27 | 32 | 0 | 0 | 24 |
| 2.2 | Escherichia | 36 | 24 | 42 | 33 | 30 | 22 | 24 |
| 3.2 | Escherichia | 34 | 22 | 35 | 30 | 29 | 23 | 22 |
| 4.2 | Escherichia | 34 | 23 | 37 | 28 | 9 | 23 | 23 |
| 5.2 | Escherichia | 37 | 23 | 40 | 31 | 30 | 24 | 23 |
| 6.2 | Escherichia | 35 | 22 | 35 | 32 | 29 | 23 | 24 |
| 8.2 | Shigella | 34 | 21 | 35 | 29 | 26 | 25 | 23 |
| 9.2 | Klebsiella | 33 | 23 | 33 | 27 | 25 | 13 | 23 |
| 10.2 | Escherichia | 37 | 22 | 38 | 34 | 30 | 0 | 25 |
| 11.2 | Shigella | 33 | 22 | 32 | 33 | 22 | 0 | 23 |
| 12.2 | Enterobacter | 35 | 22 | 38 | 29 | 32 | 11 | 23 |
| 13.2 | Escherichia | 36 | 21 | 38 | 31 | 0 | 0 | 23 |
| 14.2 | Escherichia | 35 | 23 | 38 | 31 | 31 | 24 | 23 |
| 15.2 | Enterobacter | 36 | 23 | 36 | 29 | 30 | 18 | 22 |
| 16.2 | Escherichia | 36 | 22 | 36 | 32 | 28 | 22 | 22 |
| 17.2 | Escherichia | 38 | 22 | 38 | 32 | 10 | 25 | 22 |
| 18.2 | Escherichia | 38 | 21 | 38 | 30 | 0 | 25 | 22 |
| 19.2 | Escherichia | 34 | 22 | 28 | 30 | 28 | 0 | 21 |
| 20.2 | Escherichia | 35 | 23 | 36 | 28 | 29 | 21 | 22 |
| 21.2 | Escherichia | 36 | 23 | 37 | 31 | 33 | 24 | 22 |
| 23.2 | Shigella | 36 | 0 | 0 | 30 | 0 | 24 | 18 |
| 24.2 | Citrobacter | 36 | 22 | 30 | 25 | 27 | 20 | 22 |
| 25.2 | Escherichia | 36 | 20 | 36 | 30 | 28 | 22 | 22 |
| 26.2 | Klebsiella | 32 | 22 | 32 | 21 | 20 | 10 | 25 |
| 27.2 | Escherichia | 35 | 21 | 9 | 30 | 0 | 0 | 23 |
| 28.2 | Escherichia | 35 | 25 | 41 | 31 | 30 | 21 | 22 |
| 29.2 | Escherichia | 36 | 21 | 27 | 29 | 26 | 22 | 22 |
| 30.2 | Shigella | 35 | 22 | 33 | 30 | 27 | 0 | 22 |
| 31.2 | Shigella | 37 | 23 | 36 | 30 | 28 | 24 | 25 |
| 32.2 | Escherichia | 34 | 20 | 39 | 30 | 25 | 0 | 22 |
